# Exanthematous Drug Eruption to Intravenous Iron: A Case Report

**DOI:** 10.7759/cureus.22045

**Published:** 2022-02-09

**Authors:** Shilpa S Mantri, Niraj Ballam Nagaraj, Chirag Patel, Kinjal Solanki, Haris Rana

**Affiliations:** 1 Internal Medicine, Rutgers Robert Wood Johnson Medical School, New Brunswick, USA; 2 Internal Medicine, Saint Peter’s University Hospital, New Brunswick, USA; 3 Infectious Disease, Saint Peter’s University Hospital, New Brunswick, USA; 4 Pulmonology and Critical Care, Saint Peter's University Hospital, New Brunswick, USA

**Keywords:** iron deficiency anemia (ida), maculopapular rash, delayed hypersensitivity, intravenous iron supplement, exanthematous drug eruption, type iv hypersensitivity

## Abstract

The authors present a rare case of an exanthematous drug reaction to intravenous iron. Exanthematous drug eruptions, also called morbilliform or maculopapular drug rashes, can occur in first-time drug exposures and represent a subtype of delayed-type IV hypersensitivity reactions.

This patient is a 49-year-old female with a history of iron deficiency anemia and hypothyroidism who presented to the emergency department after experiencing a diffuse whole-body maculopapular rash following ferumoxytol 510 mg intravenously received once two days prior to her presentation. A clinical examination was suspicious of an exanthematous drug eruption. The patient was treated with methylprednisolone 40 mg intravenously twice a day for three days, followed by prednisone 40 mg orally twice a day for two days with a steroid taper upon discharge. The patient’s rash resolved within five days of steroid treatment.

There is a high global prevalence of iron deficiency anemia for which intravenous iron replacement may be required. However, there is limited research addressing its adverse effects, particularly those that include delayed hypersensitivity reactions. This paper aims to alert healthcare professionals of a rare type of delayed hypersensitivity reaction to intravenous iron to better guide management in the clinical setting.

## Introduction

Approximately 25% of individuals worldwide have anemia [1]. Iron deficiency, the most common cause, is responsible for 50% of all anemias [1]. The primary treatment strategy for iron deficiency anemia is an oral iron replacement. However, intravenous (IV) iron is increasingly used for a wide range of therapeutic areas for patients with poor oral tolerance, impaired absorption, significant ongoing bleeding, or nonadherence [2-4]. Administration of IV iron is typically considered a safe procedure. However, hypersensitivity reactions of any severity to iron infusions have a reported prevalence of below 0.1% [5]. 

Overall, hypersensitivity reactions represent about one-third of all adverse drug reactions, affecting 10-20% of hospitalized patients and more than 7% of the general population [6]. Given the rarity of hypersensitivity reactions to IV iron infusions, there is a paucity of evidence about how to categorize, manage, and prevent such reactions. Many studies document incidences of anaphylactic drug reactions to IV iron. Based on a meta-analysis of published studies of iron dextran, the approximate incidence of anaphylaxis was 0.61% [7]. Data collected from 2010 to 2019 by Stojanavic et al. found that among 13,509 iron infusions, 195 infusion reactions occurred in 195 patients (1.4% of infusions) [8]. Ninety-four percent of reactions occurred during the IV infusion, with predominant symptoms of mild urticaria, angioedema, truncal myalgias, and flushing, and 6% after the infusion (between 1 hour and 5 days), characterized as an exanthematous drug eruption [8]. 

Exanthematous drug eruptions, also called morbilliform or maculopapular drug rashes, occur in 1% to 5% of first-time users of most drugs [9]. These pruritic skin reactions typically appear 4 to 21 days after a person starts taking the causative medication and are characterized by symmetrically distributed, pink-to-red macules and papules that spread rapidly and may coalesce [9]. These drug eruptions and the much rarer and more serious Stevens-Johnson syndrome (SJS), toxic epidermal necrolysis (TEN), acute generalized exanthematous pustulosis (AGEP), and drug reaction with eosinophilia and systemic symptoms (DRESS) are idiosyncratic, T-cell-mediated, delayed (type IV) hypersensitivity reactions [9-11].

While evidence of hypersensitivity reactions to IV iron commonly includes acute and anaphylactic reactions, exanthematous drug reactions to IV iron are scarcely documented and consequently less understood among health professionals. In order to better guide diagnosis and management, this report aims to further investigate exanthematous drug reactions to IV iron as encountered during a patient's case.

## Case presentation

Case description

A 49-year-old South Asian female patient, with a history of iron deficiency anemia and hypothyroidism, was admitted to the hospital with a five-day history of a diffuse whole-body rash after an IV iron infusion. On eliciting a detailed history, the patient revealed an onset of the rash, beginning on her right elbow two days after receiving an IV iron infusion (ferumoxytol 510 mg) from her primary care physician. Three days prior to admission, the patient reported that the rash had progressed to her chest, back, and bilateral upper extremities, sparing her palms. She described the rash as pruritic, erythematous, non-painful, and slightly raised in some regions. The rash was unresolved even after the patient took four diphenhydramine tablets over 24 hours. On the day of admission, the rash had progressed to her face as well as bilateral lower extremities, and it remained pruritic. She reported mild relief of symptoms after taking prednisone 8 mg, as prescribed by her primary care physician. The patient experienced a fever of 102 °F and chills, after which the patient presented to the hospital.

She did not report any new foods, diets, or medications. The patient has no known drug allergies and has taken oral iron supplements in the past. There was no one in her household with a similar rash. The patient had no associated chest pain, shortness of breath, diaphoresis, confusion, syncope, rhinorrhea, sneezing, or arthralgias. She did not report a history of sick contacts, recent travel, or hiking. She has no prior diagnoses of an autoimmune disease, inflammatory bowel disease, bleeding disorder, or sexually transmitted diseases. 

On admission, the pertinent vitals were as follows: temperature of 99 °F, blood pressure of 94/64, heart rate of 115 beats per minute, respiratory rate of 20 breaths per minute, and oxygen saturation of 99% in room air. The temperature soon after admission increased to a maximum of 103 °F. Clinical examination revealed a diffuse maculopapular rash with coalescing parts on the bilateral upper and lower extremities, chest, back, and mildly on the face. There was no lymphadenopathy, mucosal involvement or peeling, blistering, discharge, purulence, or crusting observed on the rash. She had a white blood cell count of 13,200 per mcL with a 96% neutrophil predominance, 2% lymphocytes, 2% monocytes, and 0% eosinophils and basophils. Other laboratory results were unremarkable. Inflammatory markers showed an elevated C-reactive protein of 41.4 but an erythrocyte sedimentation rate within normal limits. A chest X-ray showed no acute cardiopulmonary findings. Blood and urine cultures were negative.

Differential diagnoses 

The most probable diagnosis is delayed hypersensitivity exanthematous drug reaction to IV iron. According to the Naranjo Adverse Drug Reaction Probability Scale, a questionnaire that helps standardize assessment of causality for all adverse drug reactions, the patient's score was 5, which is suggestive of a probable adverse drug reaction [12]. The chronological manifestations of the rash and symptoms suggest that it is likely due to the IV iron. Systemic symptoms characteristic of these drug eruptions may include pruritus, fever, and elevation of acute-phase proteins, all of which were observed in this patient [9]. 

Cutaneous reactions may be difficult to distinguish from common rashes that are unrelated to medication use, particularly viral exanthems, which are also characterized by the rapid onset of widespread, symmetric eruptions of pink-to-red macules and papules with associated symptoms of fever, malaise, sore throat, and conjunctivitis. However, viral exanthems are more common in children than in adults and are typically self-limiting and mildly symptomatic [13]. Infectious mononucleosis was also in the differential diagnosis, as this viral illness may also be associated with a maculopapular rash beginning on the trunk and arms before spreading to the forearms and face [14]. This virus was tested, but the results were negative. A tick-borne panel was considered but not ordered since the patient did not report any history of recent travel, hiking, or insect bites. The patient’s rash did not show any vesicular lesions or correspond with any dermatomal pattern, making varicella-zoster and herpes simplex viruses less likely as well. 

A punch biopsy was recommended for the patient to assess cutaneous leukocytoclastic vasculitis, which is characterized by erythematous and purpuric papules predominantly on the lower extremities [9]. This diagnosis, although less probable (since the patient had a whole-body rash), could not be definitively excluded since the patient refused the biopsy. The diffuse maculopapular rash and the correlation of symptoms with the iron infusion, however, make an exanthematous drug eruption more likely.

To further delineate between other hypersensitivity reactions, the patient’s rash was not anaphylactic in origin, as the patient was not dyspneic and given the prolonged duration between the IV infusion and the onset of her symptoms. There was also no mucosal involvement, which is almost always the case in SJS and TEN, making these severe, life-threatening causes of diffuse rash unlikely [15].

The patient had no other known allergies to foods or medications, and the only new drug treatment she received was the IV iron infusion, after which her symptoms began. Because the rash was highly suspicious of an exanthematous drug eruption, the patient was treated for this delayed hypersensitivity reaction.

Treatment

The patient was treated with methylprednisolone 40 mg IV twice daily for three days and then switched to prednisone 40 mg orally twice a day for another two days. She also received diphenhydramine 25 mg orally every six hours as needed for symptom relief of pruritus. After receiving steroid treatment for five days in the hospital, the patient’s rash had completely resolved.

The patient had also received vancomycin 1 g IV and piperacillin-tazobactam 4.5 mg IV four times a day, given her leukocytosis and fever on admission. Antibiotics were discontinued after three days, as the patient’s leukocytosis and fever resolved after one day, and both blood and urine cultures were negative during the patient's stay in the hospital.

The patient was discharged with a prednisone taper, starting at 40 mg and reducing by 10 mg every three days, and was recommended to follow up with an allergist. We also recommended avoidance of IV iron infusions, because re-exposure could result in a more severe eruption [9].

## Discussion

Pathogenesis of exanthematous drug eruptions and hypersensitivity reactions to intravenous iron

Drug-related rashes are reported for nearly all prescription medications, typically at rates exceeding 10 cases per 1,000 new users [9]. Exanthematous drug eruptions are the most common drug-induced eruptions among the much rarer and more serious SJS, TEN, AGEP, and DRESS, all of which represent T-cell mediated delayed hypersensitivity reactions [9-11].

The pathogenesis of delayed hypersensitivity reactions classically involves antigen-presenting cells that present haptens, composed of the drug or its metabolite bound to a protein or peptide, to naïve T cells. These antigen-specific T cells proliferate, infiltrate the skin, and release proinflammatory mediators, such as cytokines and chemokines, which are responsible for the cutaneous manifestations and symptoms of the drug-related rash [9,16-19]. There is an alternative theory, described by the p-i (pharmacologic interaction of drugs with immune receptors) concept, whereby small-molecule drugs or their metabolites, rather than acting as antigens, activate T cells directly by binding to T-cell receptors [9,16,17]. Regardless of the pathophysiologic mechanism that elicits a T-cell mediated delayed hypersensitivity reaction, it is unclear why a minority of patients receiving a particular drug have a clinical reaction to it.

The only well-described mechanism for a hypersensitivity reaction to IV iron is for acute reactions such as anaphylaxis. For acute reactions, two documented possibilities are immunological IgE-mediated responses, for example, to the dextran component of IV iron preparations containing this molecule, and complement activation-related pseudo-allergy (CARPA) [3,20]. CARPA may be the most common mechanism of acute hypersensitivity reactions provoked by any infusion containing nanoparticles, which all IV iron preparations contain [3,21]. The final common pathway of this mechanism is likely to include activation of mast cells and basophils either directly or via anaphylatoxins (C3a and C5a) that proliferate in the blood resulting from complement activation. The secretion products of these cells, such as histamine, thromboxanes, leukotrienes, and platelet-activating factors, trigger smooth muscle contraction, increased capillary permeability, and third-spacing, resulting in bronchospasm, laryngeal edema, tachycardia, hypo- or hypertension, hypoxia, and reduced tissue perfusion [20].

There are no existing data, however, to support a mechanism other than that of the exanthematous drug eruption described above, for a delayed hypersensitivity specific to IV iron.

Risk factors for hypersensitivity reactions to intravenous iron

Factors that increase the risk or severity of hypersensitivity reactions in patients given iron infusions include a previous reaction to IV iron, fast iron infusion rate, history of other drug allergy or allergies, severe asthma or eczema, mastocytosis, severe respiratory or cardiac disease, old age, treatment with beta-blockers and/or ACE inhibitors, pregnancy (first trimester), and systemic inflammatory disease (e.g., rheumatoid arthritis and systemic lupus erythematosus) [3]. It has also been suggested that anxiety on the part of healthcare professionals giving IV drugs increases the risk of hypersensitivity reactions [22].

These risks are primarily documented in the context of anaphylactic and acute hypersensitivity reactions to IV iron [3]. Furthermore, none of these risk factors were elicited from our patient. As such, there is no clear existing evidence between these risk factors and the development of a delayed hypersensitivity reaction to IV iron.

Management of intravenous iron infusions and hypersensitivity reactions 

While there is no existing guidance on how to prevent and manage hypersensitivity reactions to IV iron, the most effective initial treatment of any eczematous drug eruption is the discontinuation of the causal medication [23].

While anyone receiving an IV iron infusion may be regarded as susceptible to a hypersensitivity reaction, some individuals are at higher risk, as discussed above. It is recommended that clinicians carefully consider whether the potential risks associated with an iron infusion are outweighed by its benefits, particularly when repeating IV iron infusions in individuals with a history of hypersensitivity reactions [3].

The treatment of exanthematous drug eruptions is largely symptomatic. The efficacy of therapies for these drug eruptions has not been evaluated in randomized trials, and their use is based on clinical experience. For symptomatic relief of exanthem and pruritus, topical corticosteroids and oral antihistamines are recommended [24]. High-potency topical corticosteroids are generally used one to two times per day for one week or until resolution, while antihistamines (preferably second-generation, non-sedating antihistamines) are continued until the pruritus subsides [24]. Systemic corticosteroids are not routinely recommended for the treatment of uncomplicated exanthematous drug eruptions. However, in patients with drug-induced rash and systemic or cutaneous symptoms suggesting a severe cutaneous reaction, as in our patient, a short course of moderate to high-dose of systemic corticosteroids (e.g., prednisone 1-2 mg/kg per day for five to seven days) may be beneficial [25]. We started our patient with IV methylprednisolone for three days prior to transitioning to oral prednisone. The patient had complete resolution of her symptoms within five days of corticosteroid treatment.

## Conclusions

There is a high global prevalence of iron deficiency anemia for which intravenous iron replacement may be clinically indicated. However, adverse yet rare hypersensitivity reactions may occur during these infusions. The rarity of hypersensitivity reactions means that there may never be a clinical trial to assess optimal therapeutic measures. Further research requires clarification of the pathogenesis of hypersensitivity reactions, risk stratification for each hypersensitivity reaction, and appropriate management of such reactions, especially that of the exanthematous drug reaction to IV iron. Our goal is to alert clinicians to this rare exanthematous manifestation to better guide treatment.
